# Normal width of the linea alba, prevalence, and risk factors for diastasis recti abdominis in adults, a cross-sectional study

**DOI:** 10.1007/s10029-021-02493-7

**Published:** 2021-10-05

**Authors:** R. L. Kaufmann, C. S. Reiner, U. A. Dietz, P. A. Clavien, R. Vonlanthen, S. A. Käser

**Affiliations:** 1 Department of Visceral and Transplantation Surgery, University Hospital of Zurich, Zurich, Switzerland; 2 Institute of Diagnostic and Interventional Radiology, University Hospital of Zurich, Zurich, Switzerland; 3 Department of General, Visceral and Plastic Surgery, Cantonal Hospital of Olten, Olten, Switzerland; 4 Department of General, Visceral, Thoracic and Vascular Surgery, Buergerspital Solothurn, Schöngrünstrasse 42, 4500 Solothurn, Switzerland; 5 Faculty of Medicine, University of Zurich, Zurich, Switzerland

**Keywords:** Diastasis recti abdominis, Rectus abdominis diastasis, Linea alba width, Interrectal distance, Normal width, Prevalence

## Abstract

**Aim:**

The prevalence and definition of diastasis recti abdominis (DRA) is under debate. This retrospective cross-sectional study evaluated the interrectal distance and the prevalence of DRA in computed tomography (CT) in an asymptomatic population.

**Materials and methods:**

Patients undergoing CT scans for suspected appendicitis or kidney stones from 01/2016 to 12/2018 were screened retrospectively to participate. A study population with equal distribution according to gender and age (18–90 years) was generated (*n* = 329 patients) and the interrectal distance was measured at six reference points.

**Results:**

DRA (defined as > 2 cm at 3 cm above the umbilicus) was present in 57% of the population. The 80th percentile of the interrectal distance was 10 mm at the xiphoid (median 3 mm, 95% confidence interval (CI) 0–19 mm), 27 mm halfway from xiphoid to umbilicus (median 17 mm, 95% CI 0–39 mm), 34 mm at 3 cm above the umbilicus (median 22 mm, 95% CI 0–50 mm), 32 mm at the umbilicus (median 25 mm, 95% CI 0–45 mm), 25 mm at 2 cm below the umbilicus (median 14 mm, 95% CI 0–39 mm), and 4 mm halfway from umbilicus to pubic symphysis (median 0 mm, 95% CI 0–19 mm). In the multivariate analysis, higher age (*p* = 0.001), increased body mass index (*p* < 0.001), and parity (*p* < 0.037) were independent risk factors for DRA, while split xiphoid, tobacco abuse, and umbilical hernia were not.

**Conclusion:**

The prevalence of DRA is much higher than commonly estimated (57%). The IRD 3 cm above the umbilicus may be considered normal up to 34 mm. To avoid over-treatment, the definition of DRA should be revised.

## Introduction

In the linea alba, collagen fibers from both sides of the abdominal wall muscle sheets cross in an interwoven pattern. This structural characteristic ensures core stability under abdominal muscle tension and allows the accommodation of intraabdominal volume by chronic separation of the medial borders of the rectus muscles. Diastasis recti abdominis (DRA) refers to an abnormal separation of the rectus abdominal muscles resulting in abdominal bulging. DRA is believed to be associated with conditions weakening the linea alba, such as multiple pregnancies [1] and obesity due to the elevated intraabdominal pressure, or previous abdominal surgery. The stretching and thinning of the linea alba favors concomitant hernia defects [2, 3] and is associated with low back pain, abdominal wall dysfunctions and decreased quality of life [4–7]. DRA can resolve spontaneously in the postpartum period, it can be corrected surgically [3, 7–11]. Conservative treatment for DRA has been proposed by various studies but no universally acceptable approach has yet been defined and proofed so far [8, 10, 12].

In clinical practice, various measuring methods for the width of the linea alba are used, such as the easy feasible “finger-width” method, tape measure and calipers [5, 13–15]. Furthermore, ultrasound is currently considered as a reliable and well-established method for pregnant and postpartum women due to the widespread clinical use and the lack of radiation [14, 16]. Computed tomography (CT) is an established method in abdominal wall diagnostics in samples with a broader range of patients [15, 17]. CT may underestimate DRA compared to intraoperative measurements [15].

*Testut and Latarjet* published normal values for the linea alba width as up to 9–14 mm cranial and up to 18 mm at the umbilicus in 1948 [18]. An interrectal distance (IRD) up to 2 cm (at 3 cm above the umbilicus) is widely considered to be physiological [2]. Nevertheless, there is a controversy regarding classification of a pathological IRD. In the literature, several classifications for DRA have been developed, which makes the comparison between studies difficult. In an ultrasound study, Beer et al. evaluated the normal linea alba width in 150 nulliparous women with a BMI < 30 kg/m^2^ in women between 20 and 45 years. Beer et al. defined DRA at three reference points with values > 15 mm at the xiphoid, > 22 mm at 3 cm above the umbilicus, and > 16 mm at 2 cm below the umbilicus (classification based on width) [19]. Based on a study on 40 cadavers, Rath et al*.* defined an IRD > 15 mm at halfway from the xiphoid to the umbilicus, > 27 mm at the umbilicus and > 14 mm at halfway from the umbilicus to the symphysis as a pathologic separation of the rectus muscles after the age of 45 years (classification based on width) [20]. Before the age of 45, Rath et al. presented 10, 27 and 9 mm as the corresponding cutoff values for IRD [20]. Nahas et al. described four anatomical types of myoaponeurotic deformities in 88 patients to classify DRA and to assess the best surgical approach (classification based on type of deformity and etiology) [21]. Using digital calipers, Chiarello et al. studied 34 cadavers between 47 and 99 years to measure the IRD 45 mm above the umbilicus, at the umbilicus and 45 mm below the umbilicus as well as to identify possible risk factors for DRA [22] using the Rath classification. Wu et al. assessed the IRD of 644 women similar to Chiarello et al. and Rath et al. and applied consequently the different cutoff values to patients below and above 45 years. Measuring with ultrasound, Mota et al*.* evaluated the “regular” IRD of 84 primiparous women at 5 cm above the umbilicus and 2 cm above the umbilicus, at the umbilicus and 2 cm below the umbilicus. During pregnancy and 6 months postpartum, Mota et al. used the 20th and 80th percentiles to define the normal width of the linea alba [16]. Recently, an expert conference confirmed DRA as the separation of more than 2 cm and re-proposed a classification of DRA in mild (< 3 cm), moderate (3-5 cm), and severe (> 5 cm) [2] similar to the classification of Ranney et al. [23]. In addition, Reinpold et al. outlined and discussed the measurement positions for DRA as Rath et al., Mota et al., and Beer et al. proposed [2]. Interestingly clinical symptoms and findings of physical examination are rarely discussed in the context of definition of DRA, bearing the potential bias of suggesting treatment for a body-part mean measurement.

Many publications showed an elevated prevalence of DRA in multiparous women and obese men due to the stretching and thinning of the linea alba and the abdominal wall [16, 24–27]. Janes et al. described that linea alba width increases significantly with parity, in particular after the first and second pregnancy [1]. Midline hernia, umbilicus hernia, and groin hernia are often concomitants [2, 3, 23]. Moesbergen et al. describes the correlation of DRA and the width of aorta [28]. Proposed and researched risk factors were weight gain during pregnancy, delivery mode, baby’s birth weight, benign joint hypermobility syndrome, split xiphoid, heavy lifting, general exercise training, lumbo-pelvic pain, urogenital dysfunctions, level of abdominal, and pelvic floor muscle [29, 30].

The available data are consistently limited to pregnant women, postpartum women, obese patients, and cadavers. The prevalence of DRA in the standard population throughout all adult ages and both gender has yet not been described [31, 32]. Thus, to our knowledge, a definition of DRA based on a normalized adult population is missing. The yet available measurements of the linea alba should not be generalized to the asymptomatic population.

The present study aims at filling this gap. In a general adult population adjusted to age and gender, this retrospective study assessed the extent of IRD at six reference points and the prevalence of DRA. CT scans done for suspected appendicitis or kidney stones were evaluated combining the classifications of Beer et al., Rath et al., and Ranney et al. This study contributes to a better understanding of the condition and a recognized universal classification for the diagnosis of DRA.

## Materials and methods

### Participants

This was a retrospective cross-sectional study analyzing men and women from 18 to 90 years. All patients undergoing a CT for suspected appendicitis or kidney stones at the Institute of Diagnostic and Interventional Radiology at the University of Zurich from January 2016 to December 2018 were considered to participate in the study. For each age, the prerequisite was to analyze at least two males and two females. Inclusion criteria were male and female patients between 18 and 90 years. Exclusion criteria were missing general informed consent, history of previous laparotomy or rectus diastasis repair and previous laparoscopic hernia repair. A total of 329 patients (155 women, 174 male) were included and analyzed.

### Instrumentation and procedures

**Fig. 1:**
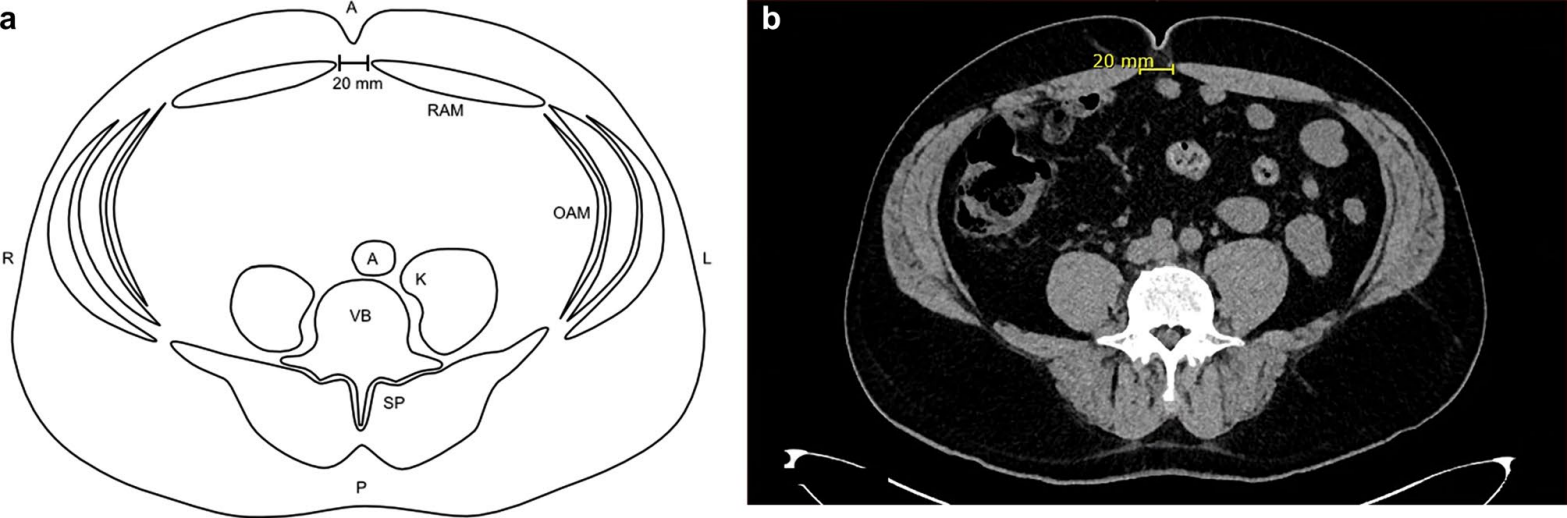
**a**, **b** Measurement of the IRD showed 20 mm at the umbilicus on a CT scan^5^. ^5^*A* aorta, *K* kidney, *OAM* oblique abdominal muscles, *RAM* rectus abdominis muscle, *SE* spinal erectors, *VB* vertebral body

CT as an established reliable method is used to assess DRA [15, 17]. Static CT images were generated using a dual-source CT (Definition AS, Somatom Definition Flash and Somatom Force, Siemens Healthineers). Data acquisition was done with the participants in a supine resting position with straight legs and arms alongside the body, in an inspiration breath hold, but without explicit Valsalva maneuver. Figure 1 shows the interrectal distance in a CT scan and in a graphic for better visualization. The measurements of the interrectal distance were all performed by the same investigator with the software AGFA IMPAX 6.0. An integrated ruler indicated the measurement and the accuracy was at the millimeter level.

### Interrectal distance measurements

**Fig. 2:**
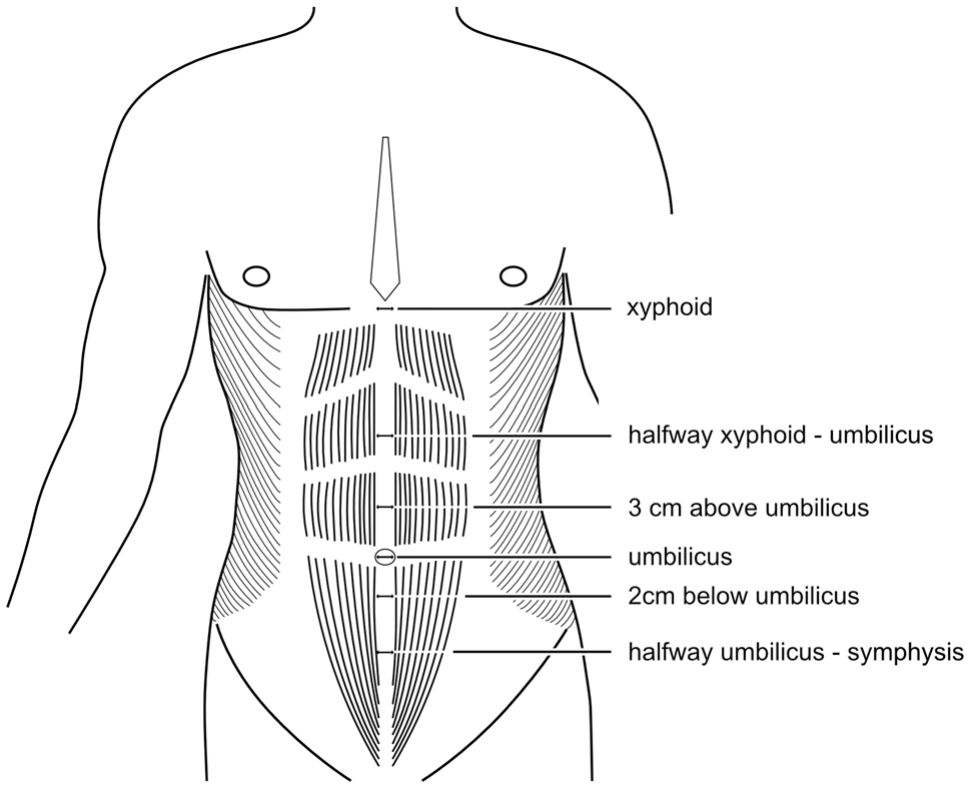
Reference points for the measurement of the linea alba width

As depicted in Fig. 2, six representative locations on the linea alba were defined to guarantee valid measurements, referring to Beer et al. [19] and Rath et al. [20]. The IRD was measured at the xiphoid, halfway from the xiphoid process to the umbilicus, 3 cm above the umbilicus, at the umbilicus, 2 cm below the umbilicus, and halfway from the umbilicus to the pubic symphysis.

DRA is considered as a pathological separation of more than 2 cm by studies such as Beer et al. (> 22 mm at 3 cm above the umbilicus) [19], Rath et al. (> 10 mm above and > 27 mm at the umbilicus) [20], Mota et al. (> 28 mm at 2 cm above the umbilicus) [16] and Chiarello et al. (> 23 mm at 4.5 cm above the umbilicus) [33]. Based on an expert conference, Reinpold et al. stated 2019 that DRA is widely considered as a separation of more than 2 cm [2]. Therefore, in the present study, the cutoff value for DRA was set at an IRD of > 20 mm at the measurement point at 3 cm above the umbilicus. At the level of the umbilicus, the investigator assessed whether an umbilical hernia was present.

### Sociodemographic characteristics

Based on published studies [26, 29, 34], possible risk factors related to the presence of DRA were identified. Sociodemographic parameters included size (m), weight (kg), BMI (kg/m^2^), tobacco use (py), and the number of pregnancies, mainly obtained based on anesthesia protocols or extended medical history of digitized patient records. The information was anonymized and collected in an excel sheet.

### Statistics

Statistical analysis was performed with the Stata 10 statistics program (StataCorp LLC 4905 Lakeway Drive, College Station, Texas, USA). Results are presented as median and range or mean and standard deviation whenever justified. Categorical data were analyzed with the two-sided Fisher’s exact test while analysis of continuous data was performed with the Wilcoxon rank-sum test in binary variables and linear regression in continuous variables. A *p* value of < 0.05 was considered statistically significant. Significant variables identified in the univariate analysis were subjected to a logistic regression analysis, defining the odds ratio (OR), standard deviation (SD) and the 95% confidence interval (CI).

### Ethics

Regarding general consent, every patient of the University Hospital Zurich receives a declaration of consent for the further use of health-related personal data and biological material for research upon admission, which he or she can confirm or refuse at any time. The majority of the patients had approved written general consent, whereas patients with unknown consent received a letter explaining the study and a template for a possible rejection of the use of their data in this particular study. The Cantonal Ethics Committee of Zurich, Switzerland, approved the study (BASEC ID 2019-00110).

## Results

After screening 603 patients, 274 patients had to be excluded: 55 met the exclusion criteria, 91 dismissed general consent, 4 dismissed individual consent and 124 had missing data. Thus, 329 patients, of whom 155 (47%) are women, and 174 (53%) are men, were included in the cross-sectional study. For each age from 18 to 90 years, at least two females as well as two males and on average 4.5 participants (range 3–9) were analyzed. Due to retrospective rejection of consent, one age category only included three patients. The mean age of the 329 participants was 54.4 years (range 18–90) and the mean body mass index (BMI) was 26.2 kg/m^2^ (range 16–63). 107 (33%) of them were smokers, and their mean tobacco use was 25.4 pack years. Of 155 women in the sample, 57 were nulliparous whereas 86 had at least 1 child and in 12 patients, these data were not available.

**Fig. 3:**
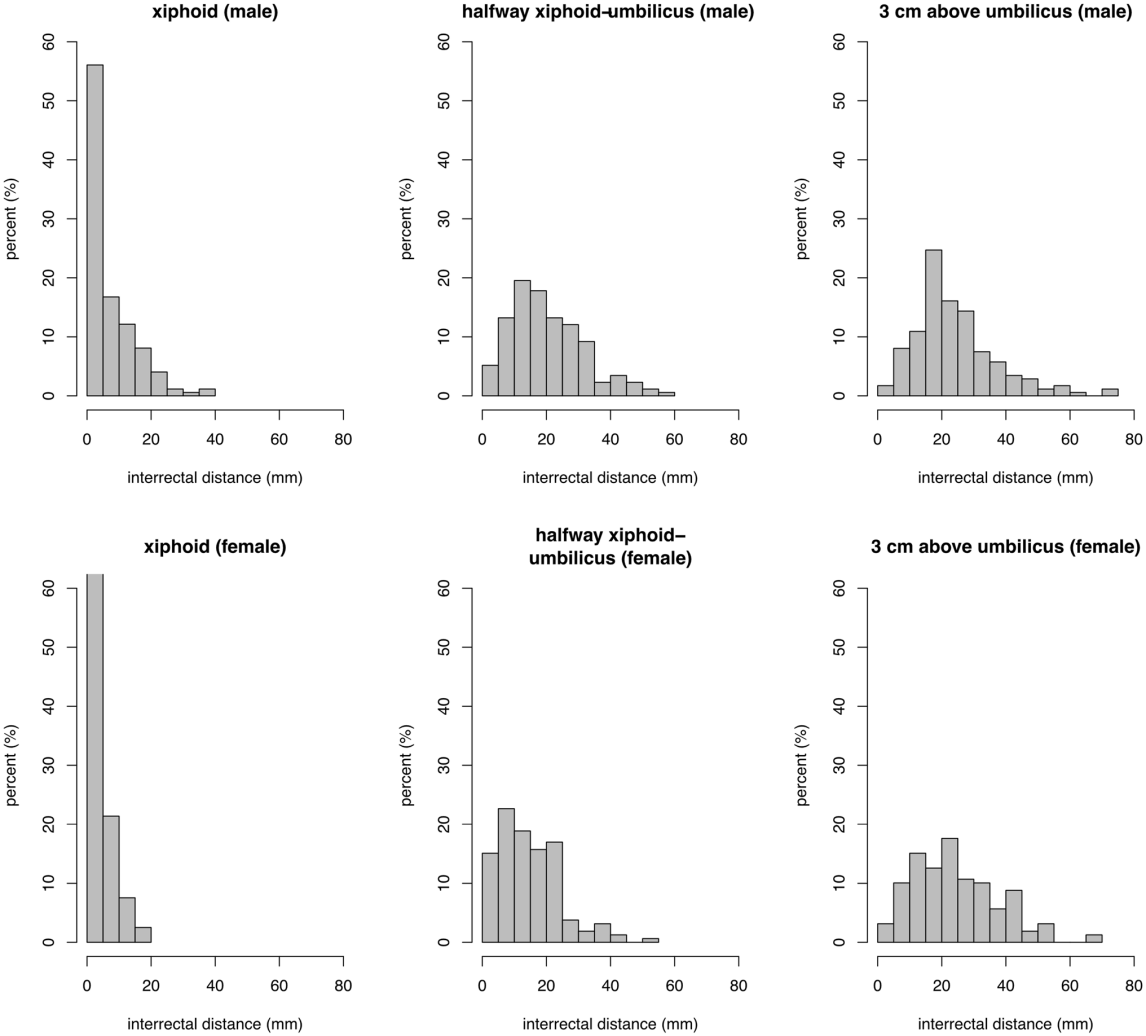
Distribution of the interrectal distances at different reference points above the umbilicus

**Fig. 4:**
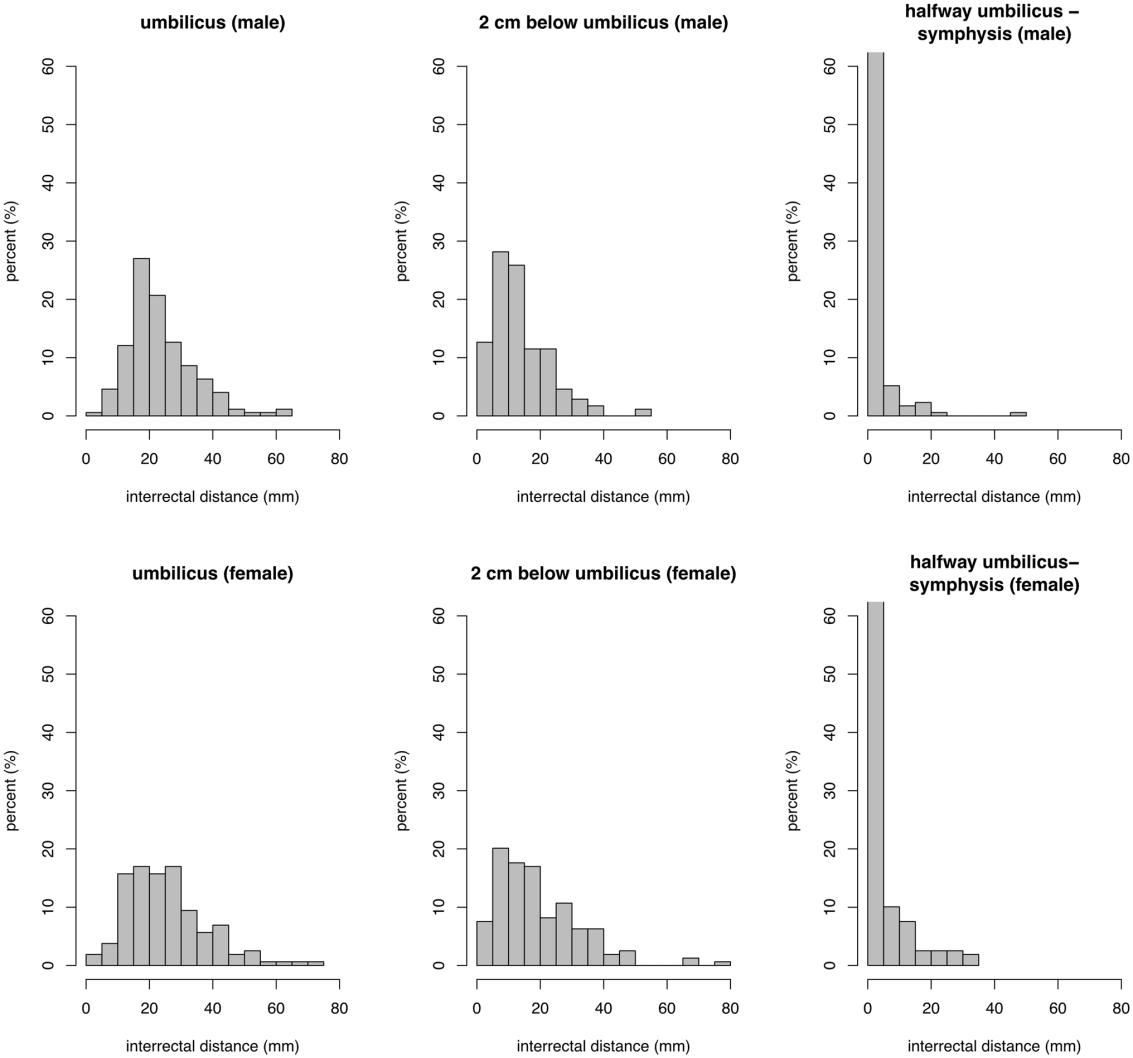
Distribution of the interrectal distances at different reference points at and below the umbilicus

The distribution and extent of IRD for men and women each at the six reference points are depicted in Figs. 3 and 4. The 20th, 50th, and 80th percentiles are displayed in Table 1. The data reveals that the width of the linea alba can be considered normal in the general population up to 10 mm at the xiphoid, up to 27 mm halfway from xiphoid to umbilicus, up to 34 mm at 3 cm above the umbilicus, up to 32 mm at the umbilicus, up to 25 mm at 2 cm below the umbilicus, and up to 4 mm halfway from umbilicus to pubic symphysis. Table 1 shows the mean and standard deviation for the six reference points. Table 2 shows the mean IRD at the six reference points for each age group. The variance of IRD is most marked at 3 cm above the umbilicus. The mean IRD at 3 cm above the umbilicus in the general population is 22 mm. The 5% cutoff value of IRD in this sample was 50 mm at 3 cm above the umbilicus.

**Table 1:** Percentiles and mean ± SD (mm) of the linea alba width at the six abdominal reference points

	95th	90th	80th	70th	60th	50th	40th	30th	20th	10th	Mean	± SD	Min	Max
Xiphoid	19	15	10	7	5	3	2	0	0	0	6	6.91	0	40
X–U	39	32	27	23	19	17	14	11	9	6	18	11.08	0	57
3 cm AU	50	43	34	29	25	22	20	17	14	10	25	12.88	0	71
Umbilicus	45	41	32	29	25	23	20	18	16	12	25	11.64	0	75
2 cm BU	39	31	25	20	16	14	11	10	8	5	17	11.68	0	77
U–S	19	12	4	0	0	0	0	0	0	0	3	6.67	0	46

*X–U* halfway xiphoid–umbilicus, *AU* above umbilicus, *BU* below umbilicus, *SD* standard deviation, *U–S* halfway umbilicus–symphysis pubis

**Table 2:** Mean of the linea alba width at the six abdominal reference points according to age groups

Age (y)	All	18–29	30–39	40–49	50–59	60–69	70–79	80–89
Xiphoid	6	3	4	4	6	4	9	9
X–U	18	11	16	16	18	18	23	24
3 cm AU	25	15	22	23	26	27	30	30
Umbilicus	25	17	22	24	27	28	27	28
2 cm BU	17	10	14	16	19	17	18	22
U–S	3	0	2	1	3	2	4	8

*X–U* halfway xiphoid–umbilicus, *AU* above umbilicus, *BU* below umbilicus, *U–S* halfway umbilicus–symphysis pubis, *y* year

**Fig. 5:**
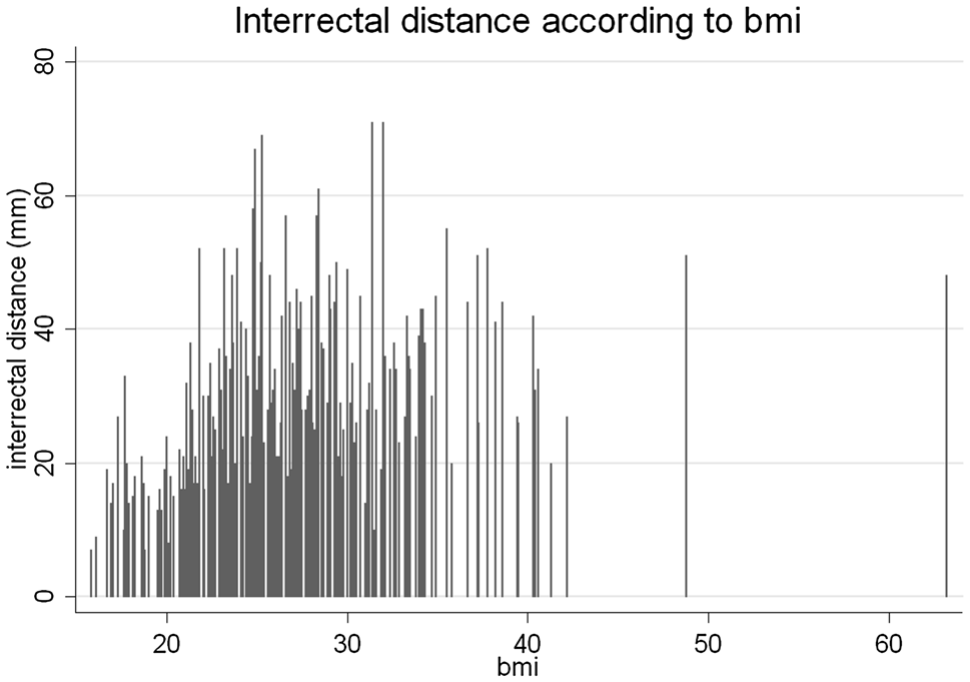
IRD (mm) according to BMI (kg/m^2^)

**Fig. 6:**
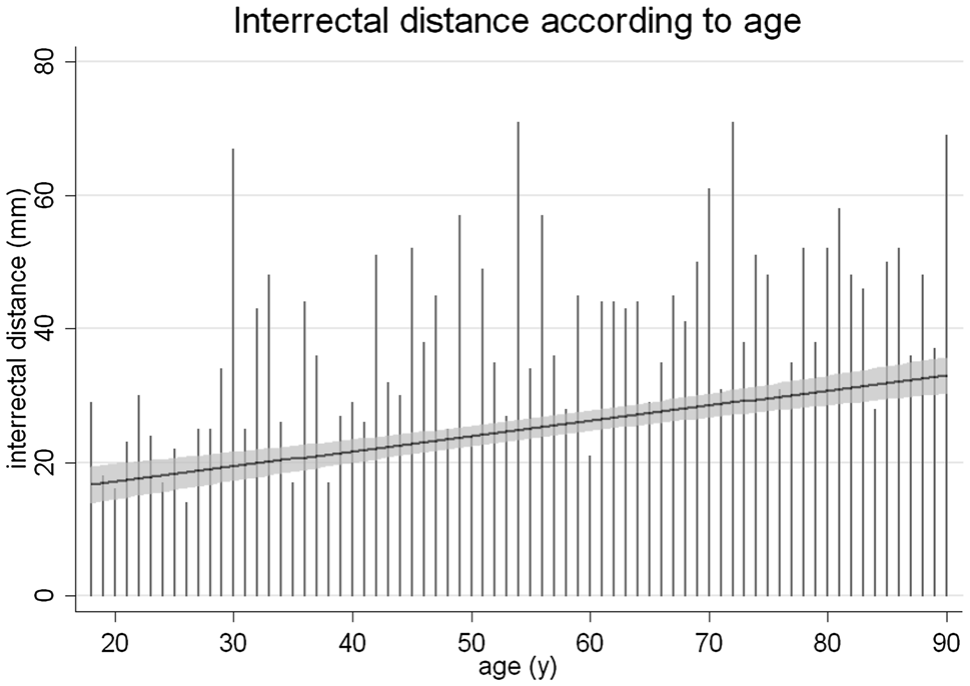
IRD (mm) according to age (years) with its linear regression of the 95% CI

The prevalence of IRD > 2 cm according to the current definition of DRA at 3 cm above the umbilicus with regard to age and in general is presented in Table 3. Table 4 shows the results of univariate analysis of possible risk factors for DRA. Hereby age, BMI, split xiphoid, parity, and umbilical hernia were significantly correlated with DRA. In multivariate analysis, only higher age, increased BMI and parity remained significantly correlated with DRA as shown in Table 5. Figures 5 and 6 show the distribution and correlation of DRA with age and with BMI, respectively.

**Table 3:** Prevalence of DRA at 3 cm above the umbilicus at different ages with the current definition of IRD over 20 mm

Age (y)	All	18–29	30–39	40–49	50–59	60–69	70–79	80–89
Prevalence	57%	24%	61%	53%	65%	67%	72%	72%
Degree								
Minor	47%	83%	63%	52%	50%	45%	35%	33%
Moderate	44%	17%	32%	37%	44%	52%	50%	53%
Pronounced	9%	0%	5%	11%	6%	3%	15%	14%

**Table 4:** Risk factors compared to adults with and without DRA at ages between 18 and 90 years

Variable	DRA (*n* = 185)	No DRA (*n* = 144)	*p*-value
Age (years): mean (± SD)	60.5 (± 19.0)	46.5 (± 20.2)	** < 0.0001**
BMI (kg/m^2^): mean (± SD)	28.3 (± 5.7)	23.5 (± 3.9)	** < 0.0001**
Split xiphoid: *n*	91 (49%)	46 (32%)	**0.032**
Gender (*n*)—paired analysis			0.556
Males	23	32	
Nulliparous women	19	36	
Parity (*n*)			**<0.001**
Nulliparous women	20	37	
Primi/multiparous women	69	17	
Umbilical hernia: *n*	34	9	**0.001**
Smoking: *n* (%)	56 (30%)	51 (35%)	0.404
Pack years: mean	28.6	21.8	0.685

Bold values indicate statistically significant *p* values (*p* < 0.05)

*BMI* body mass index, *DRA* diastasis recti abdominis, *SD* standard deviation

**Table 5:** Results of logistic analysis of OR with 95% CI to predict possible risk factors associated with the presence of DRA in adults at ages between 18 and 90 years

DRA	OR	SD	*z*-statistic	*p* value	95% CI for OR	
Age	1.028	0.008	4.47	**0.001**	1.012	1.045
BMI	1.299	0.062	5.46	** < 0.001**	1.182	1.426
Split xiphoid	1.821	0.590	1.85	0.064	0.965	3.435
Parity	1.383	0.215	2.08	**0.037**	1.019	1.877
Umbilical hernia	1.485	0.485	1.21	0.227	0.782	1.817

Bold values indicate statistically significant *p* values (*p* < 0.05)

*OR* odds ratio, *SD* standard deviation, *CI* confidence interval

## Discussion

The present study revealed that the mean linea alba width was 22 mm (± SD 12.88) in an adult population of all age groups. The prevalence of diastasis recti abdominis at 3 cm above the umbilicus according to the current definition was as high as 57%. Age, BMI and parity were statistically significant risk factors for DRA.

The German Hernia Society (DHG) and the International Endohernia Society (IEHS) recently published a definition and a classification of DRA based on a consensus conference. Hereby, DRA was defined as a separation of the rectus muscles of more than 2 cm with three categories (< 3 cm, 3–5 cm, and > 5 cm) [2, 23]. The chosen cutoff values in this present study followed this proposal and extended some measurement points according to Beer et al. and Rath et al. [2, 19, 20]. Analyzing not only a restricted patient group but assessing a standardized population, this study aimed at assessing the IRD in a general population to improve the reliability of the definition of DRA.

This study showed that age, BMI, and parity are significant risk factors for the presence of DRA. Since obesity leads chronically and pregnancy leads temporary to an elevated intraabdominal volume and pressure, it is obvious why a greater BMI and parity is associated with increased IRD. The association of DRA with increasing age correlates with results from Spitznagle et al. [35] but is in contrast with Wu et al. [36]. However, the study by Wu et al. differ in the study design and the evaluation criteria of DRA using the Rath classification and separate cutoff values for DRA in patients under 45 years and over 45 years. Therefore, the threshold to diagnose DRA in young females was lower than in older females. To prove young age as a risk factor, *Wu *et al*.* compared the group of elderly women (≥ 60 years) to young women (< 45 years). This categorical analysis is in contrast with the continuous analysis of this study. Regarding participant numbers in the study by Wu et al., young women are underrepresented (< 45 years, *n* = 116) compared to the elderly (> 60 years, *n* = 321) in contrast with the linear distribution of the age of patients in this study. Racial differences in connective tissue might be another aspect for differences in age as a risk factor for DRA [35, 36]*. *Wu et al*.* explained the differences with the higher likelihood of pregnancy in young age and the possibility of suffering DRA reduces with time through longer recovery.

Interestingly no association with DRA was found concerning gender, split xiphoid, tobacco use, and umbilical hernia in this general population. This finding is consistent with literature [26, 29, 34, 35, 37].

When assessing the normal linea alba width and the prevalence of DRA, the question arises whom to include in a study. Many studies focused on women during pregnancies and postpartum [16, 25–27, 38–40], on women with urogenital issues and low back pain [35, 41] or on cadavers [20, 33]. To our knowledge, the published studies on IRD or DRA included only specific populations. Thus, the present study gives a more reliable and more valid view on the prevalence of DRA in the general population. In postpartum women, Mota et al. showed a prevalence as high as 39% [26], whereas Sperstad et al. found 32.6% [29] and Turan et al. only 19.7% [39]. In patients seeking urogynecological examination, Spitznagle et al. presented a prevalence of 52% [35]. Wu et al. presented a prevalence of 28.4% in adult females [36]. Only the study of Chiarello et al. showed a high prevalence of 74% DRA in a cadaver study in an aged population. The now reported prevalence of 57% in a standardized population aged between 18 and 90 years and in both genders is a new relevant finding. This finding should be taken into account when discussing future cutoff values of DRA, perhaps even a new definition of DRA is warranted.

The analyzed standardized sample is based on patients attending medical care for kidney stones and appendicitis at the University Hospital of Zurich. On purpose, acute diseases with often performed CT scans not associated with widening of the linea alba and prevalent throughout all ages were chosen to identify the study population. Indeed, the anthropometric parameters of this study suggest that the study population is equivalent to the general Swiss population: mean BMI (26.2 vs. 25 kg/m^2^), smokers (33% vs. 27%), and parity (1.2 vs. 1.5 children/women). The remaining small differences can be explained by the linear distribution of the age of patients in this study.

### Limitations

Retrospectively acquiring data from medical history instead of questionnaires or direct patient contact may lead to incomplete and biased data. Patients had to declare their height and weight among other disclosures before a CT scan. Therefore, height, weight and BMI were up to date. However, other patients' characteristics might be incomplete due to the retrospective design of this study.

The study cohort does not give any information on clinical symptoms potentially related to DRA and thus does not allow direct conclusions regarding the clinical relevance of DRA in this population.

CT is considered a valuable method to assess the morphology of the abdominal wall. As the IRD is underestimated in CT scans, the results are not fully comparable with other methods of measuring IRD. However, the expected bias would be towards lower values [13, 15]. The study presents a large sample size of 329 subjects, compared to other publications in this field mainly with lower sample sizes [5, 20, 26, 29, 33, 39, 42].

As there are various classifications on DRA and scant information for the normal width of the linea alba, more population-based studies are warranted. A critical reevaluation of the morphological definition amended with new criteria of symptoms and findings at physical examination will need to be addressed in prospective studies to eventually guide surgeons and patients in the task of surgical indications. DRA is associated with large BMI. This is why DRA will possibly develop to a more significant burden in the future.

## Conclusion

In the general population, the prevalence of DRA is as high as 57%. The IRD at 3 cm above the umbilicus may be considered normal up to 34 mm using the 80th percentile which exceeds all current cutoff values for DRA by far. Age, BMI, and parity are independent risk factors for DRA. To avoid over-treatment, the definition of DRA should be revised and amended with criteria such as symptoms and findings at physical examination.

## Data Availability

The original dataset is stored by the corresponding author.

## Appendix

See Tables 1, 2, 3, 4 and 5.
